# Variations in central venous oxygen saturation and central venous-to-arterial carbon dioxide tension difference to define fluid responsiveness: a prospective observational study

**DOI:** 10.3389/fcvm.2025.1628380

**Published:** 2025-11-13

**Authors:** Xiaoyang Zhou, Hanyuan Fang, Chang Xu, Jianneng Pan, Hua Wang, Tao Pan, Zhaojun Xu, Bixin Chen

**Affiliations:** 1 Department of Intensive Care Medicine, Ningbo No.2 Hospital, Ningbo, Zhejiang, China; 2 Department of Emergency, Ningbo Yinzhou No.2 Hospital, Ningbo, Zhejiang, China

**Keywords:** central venous oxygen saturation, central venous-to-arterial carbon dioxide tension difference, fluid responsiveness, volume expansion, oxygen consumption, oxygen delivery, mechanical ventilation, hypotension

## Abstract

**Introduction:**

Fluid-induced variations in central venous oxygen saturation (*Δ*ScvO_2_) and central venous-to-arterial carbon dioxide tension difference [*Δ*P(cv-a)CO_2_] have been proposed to define fluid responsiveness. This study aimed to determine whether their diagnostic accuracies are affected by baseline values or oxygen consumption (VO_2_) responsiveness.

**Materials and methods:**

This prospective observational study enrolled mechanically ventilated patients with circulatory shock. Hemodynamic variables and blood gas analysis were measured before and after a fluid challenge. Fluid responsiveness and VO_2_ responsiveness were defined as a ≥10% increase in cardiac index and VO_2_, respectively. The Spearman's rank correlation coefficient (rho) was computed to evaluate the association between variables. The diagnostic accuracy was assessed using the area under the receiver operating characteristic curve (AUC), with subgroup analyses based on baseline ScvO_2_ and P(cv-a)CO_2_ values and VO_2_ responsiveness.

**Results:**

Out of 58 patients enrolled, 30 were fluid responders. The fluid-induced changes in cardiac index were significantly correlated with *Δ*ScvO_2_ (rho = 0.36, *P* = 0.006) and *Δ*P(cv-a)CO_2_ (rho = −0.35, *P* = 0.006). *Δ*ScvO_2_ and *Δ*P(cv-a)CO_2_ defined fluid responsiveness with AUC values of 0.76 [95% confidence interval (CI): 0.63–0.86, *P* < 0.001] and 0.72 (95% CI: 0.59–0.83, *P* < 0.001), respectively. A cutoff value of 5% for *Δ*ScvO_2_ and −2 mmHg for *Δ*P(cv-a)CO_2_ yielded positive predictive values of 88% and 75%, and negative predictive values of 63% and 61%, respectively. The gray zones for *Δ*ScvO_2_ (−3 to 4.6%) and *Δ*P(cv-a)CO_2_ (−2.7 to 1 mmHg) comprised 51.7% and 48.3% of the patients, respectively. In the subgroup analyses, *Δ*ScvO_2_ potentially exhibited better accuracy for assessing fluid responsiveness in VO_2_ non-responders (AUC of 0.91, 95% CI: 0.78–0.98; 40 patients) and patients with a baseline ScvO_2_ < 70% (AUC of 0.84, 95% CI: 0.67–0.95; 32 patients). Meanwhile, the diagnostic accuracy of *Δ*P(cv-a)CO_2_ was slightly improved in VO_2_ non-responders (AUC of 0.78, 95% CI: 0.62–0.90; 40 patients) and patients with a baseline P(cv-a)CO_2_ ≥ 6 mmHg (AUC of 0.78, 95% CI: 0.62–0.90; 39 patients).

**Conclusion:**

*Δ*ScvO_2_ and *Δ*P(cv-a)CO_2_ are potential indicators of fluid responsiveness in mechanically ventilated patients with circulatory shock, especially those with abnormal baseline values or VO_2_ unresponsiveness.

## Introduction

In the intensive care unit (ICU), volume expansion represents the most commonly used measure to correct hypotension and hypoperfusion, aiming to improve oxygen delivery (DO_2_) by increasing cardiac output (CO), thereby ameliorating tissue perfusion. Whether volume expansion can elevate CO depends on whether the heart functions on the steep portion of the Frank-Starling curve, indicating fluid responsiveness (1). In recent years, the study of oxygen and carbon dioxide (CO_2_) metabolism has gained attention for assessing fluid responsiveness (2–4). From a physiological perspective, oxygen and CO_2_ metabolism are closely related to blood flow, as it provides oxygen to the tissues and removes CO_2_ produced by them (5, 6).

In recent studies, variations in central venous oxygen saturation (ScvO_2_) (*Δ*ScvO_2_) and central venous-to-arterial carbon dioxide tension difference (P(cv-a)CO_2_) [*Δ*P(cv-a)CO_2_] during volume expansion have been confirmed to assess fluid responsiveness (2–4). Indeed, according to the Fick principle, *Δ*ScvO_2_ during volume expansion can track changes in CO if oxygen content and oxygen consumption (VO_2_) remain stable, and *Δ*P(cv-a)CO_2_ during volume expansion is inversely proportional to the CO changes under consistent CO_2_ production (5–8). However, VO_2_ and CO_2_ production may not always remain unchanged during volume expansion due to the VO_2_/DO_2_ dependency phenomenon and anaerobic CO_2_ production (9–11). Furthermore, whether the diagnostic accuracies of *Δ*ScvO_2_ and *Δ*P(cv-a)CO_2_ depend on their baseline values remains unknown, even though the baseline ScvO_2_ and P(cv-a)CO_2_ seem unable to identify fluid responsiveness (2). This study aimed to determine whether their diagnostic accuracies are affected by baseline ScvO_2_ and P(cv-a)CO_2_ values or the fluid-induced VO_2_ responsiveness.

## Materials and methods

This prospective observational study was conducted in the ICU of Ningbo No. 2 Hospital from January 2024 to December 2024. It was part of a study program registered with the Chinese Clinical Trial Registry (ChiCTR2100053665) and approved by the local institutional ethics committee (YJ-NBEY-KY-2022-147-01). This manuscript adheres to the applicable STROBE guidelines (12). Written informed consent was obtained from the patients’ relatives. This study was conducted in compliance with the Declaration of Helsinki.

### Patients

The eligible subjects were mechanically ventilated adults (age ≥ 18 years) with circulatory shock and without spontaneous respiratory efforts, for whom the attending physician decided to perform a fluid challenge, where circulatory shock was defined as the presence of one or more of the following signs: 1) systolic arterial pressure < 90 mmHg, mean arterial pressure < 65 mmHg, or requiring vasopressor administration; 2) skin mottling; 3) urine output < 0.5 mL/kg/h for ≥ 2 h; 4) lactate level > 2 mmol/L. Patients would be excluded if they met the following criteria: no indwelling arterial or central venous catheterization, aortic valve surgery, equipped with extracorporeal membrane oxygenation or a pacemaker, contraindications to fluid challenge, poor echogenicity, atrial fibrillation, refractory shock expected to die within 24 h, or decline to participate.

### Study protocol

**Figure 1:**
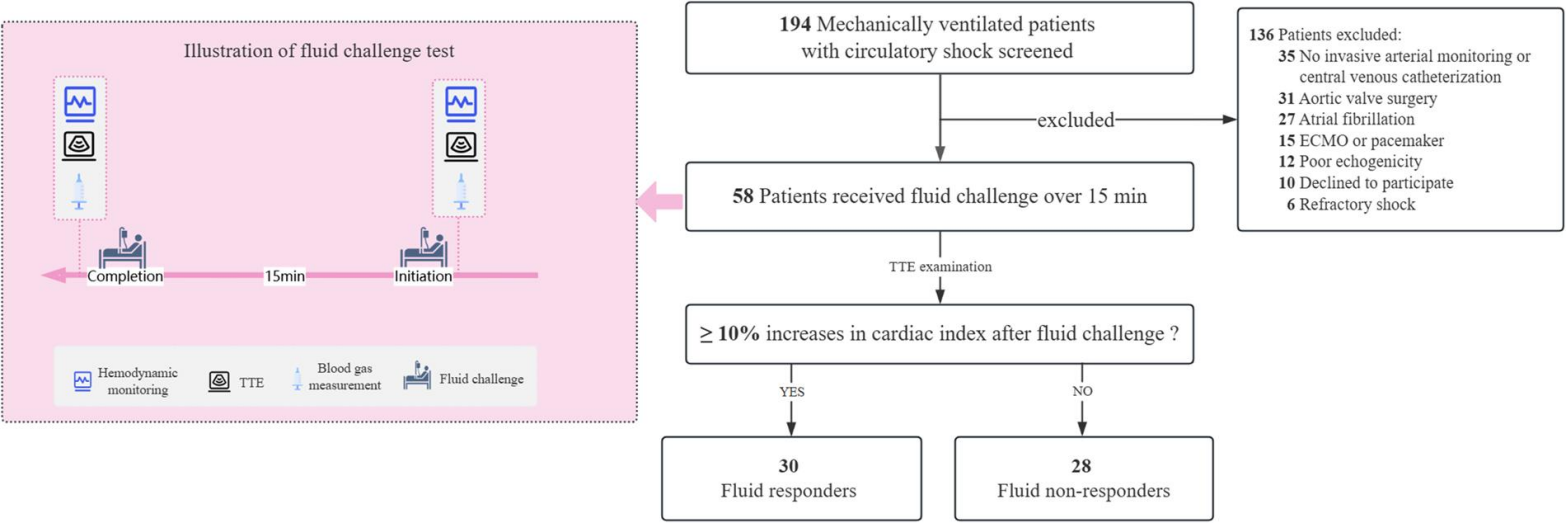
Illustration of study selection and fluid challenge test. ECMO, extracorporeal membrane oxygenation; TTE, transthoracic echocardiography.

All eligible patients received invasive radial arterial monitoring and central venous catheterization, with the catheter tip positioned in the superior vena cava or the right atrium. Pressure calibration was performed in the supine position, with pressure transducers zeroed at the phlebostatic axis, a position corresponding to the right atrium's level (the midpoint of the fourth intercostal space at the midaxillary line) (13). A pressure-controlled ventilation mode was set, and sedative and analgesic medications were continuously administered to avoid spontaneous breathing efforts. Once enrollment was confirmed, a baseline set of hemodynamic variables was measured, and transthoracic echocardiography (TTE) was performed. Meanwhile, arterial and central venous blood gases were simultaneously sampled and analyzed using a GEM Premier 3,500 blood gas analyzer (Instrumentation Laboratory Company, Bedford, MA, USA). Immediately after that, a fluid challenge test was conducted by administering a pressurized bolus of 500 mL of Ringer's solution over 15 min in the 45° semi-recumbent position. Immediately after the fluid challenge, a second set of the above measurements was taken. During the study period, no adjustments were made to body position, ventilator settings, vasopressors, inotropes, or sedative and analgesic drugs. Figure 1 illustrates the detailed process of this study.

### Data collection

We collected demographic information (including age, gender, body mass index, and comorbidities), causes of shock, ventilator-related parameters (including tidal volume, positive end-expiratory pressure, driving pressure, respiratory rate, and fraction of inspired oxygen), acute physiology and chronic health evaluation II score, sequential organ failure assessment score, sedative and analgesic drugs, and vasoactive agents at the time of enrollment. We measured and recorded hemodynamic variables [including heart rate (HR), central venous pressure, systolic arterial pressure, diastolic arterial pressure, and mean arterial pressure], echocardiographic parameters [including stroke volume (SV), cardiac index, and velocity-time integral (VTI)], arterial and central venous blood gases parameters (including potential of hydrogen (PH), arterial lactate level, arterial partial pressure of carbon dioxide (PaCO_2_), arterial oxygen saturation (SaO_2_), central venous partial pressure of carbon dioxide (PcvCO_2_), and ScvO_2_), and oxygen-CO_2_ derived variables (including DO_2_, VO_2_, and P(cv-a)CO_2_) at baseline and after the fluid challenge. The hemoglobin (Hb) concentration was measured together with arterial blood gas analysis using the GEM Premier 3,500 blood gas analyzer (Instrumentation Laboratory Company, Bedford, MA, USA). Patients were followed up until ICU discharge.

SV was computed as: VTI × LVOT area, where VTI was measured via continuous Doppler transaortic flow on an apical five-chamber view, and LVOT area was calculated as π × (LVOT diameter/2)^2^ (LVOT refers to the left ventricular outflow tract). Then, cardiac index was calculated as (SV × HR)/body surface area. TTE examination was conducted with the CX50 ultrasound system (Philips Medical System, Suresnes, France), which was performed by an independent ICU physician who was blinded to the study outcomes. The representative value of echocardiographic parameters was obtained by averaging three consecutive measurements, regardless of the respiratory cycle.

### Definition

*Δ*ScvO_2_ and *Δ*P(cv–a)CO_2_ induced by volume expansion were calculated as absolute changes, that is, subtracting the baseline value from the value after volume expansion. The fluid-induced changes in cardiac index and VO_2_ were calculated as relative changes: (the value after fluid infusion – the baseline value)/ the baseline value×100%. Fluid responsiveness and VO_2_ responsiveness were defined by a ≥10% increase in cardiac index and VO_2_ in response to volume expansion, respectively. According to the Fick principle, DO_2_ was calculated as (SV × HR) × (1.34 × Hb × SaO_2_ + 0.003 × PaO_2_), and VO_2_ was calculated as (SV × HR) × [(1.34 × Hb × SaO_2_ + 0.003 × PaO_2_) − (1.34 × Hb × ScvO_2_ + 0.003 × PcvO_2_)], where PaO_2_ is the arterial oxygen tension, and PcvO_2_ is the central venous oxygen tension.

### Statistical analysis

Statistical analyses were performed using SPSS version 17.0 (IBM, New York, USA). The normal distribution of continuous variables was assessed using the Kolmogorov–Smirnov test. Normally distributed variables are presented as means ± standard deviation (SD), and skewed variables are reported as medians with interquartile ranges (IQR). Categorical variables are described as frequencies and percentages. For the continuous data, either the Student's t-test or the Mann–Whitney test was used for inter-group comparison, depending on the data distribution, and the Student's paired t-test was applied for the intra-group comparisons. The Chi-squared test or Fisher's exact test was utilized to compare categorical variables. The Spearman's rank correlation coefficient (rho) was computed to evaluate the association between the fluid-induced changes in cardiac index and *Δ*ScvO_2_ and *Δ*P(cv–a)CO_2_.

The MedCalc Statistical Software (MedCalc Software bvba, Ostend, Belgium) was employed to construct ROC curves for assessing the diagnostic accuracy of *Δ*ScvO_2_ and *Δ*P(cv–a)CO_2_ for fluid responsiveness. The optimal cutoff value was determined by maximizing the Youden index, while taking into account the smallest detectable differences (SDD) of ScvO_2_ (±3%) and P(cv–a)CO_2_ (±2 mmHg), as previously reported (14). Additionally, the gray zone approach was used to avoid the binary constraint of a “black-or-white” decision of the optimal cutoff value (15). We calculated the gray zone for *Δ*ScvO_2_ and *Δ*P(cv–a)CO_2_ based on values that did not allow for having 10% of diagnosis tolerance (i.e., a sensitivity of <90% or a specificity of <90%) (15). To identify potential factors affecting the diagnostic accuracy, we performed subgroup analyses based on baseline ScvO_2_ value (≥70% or <70%), baseline P(cv–a)CO_2_ value (≥6 mmHg or <6 mmHg), and fluid-induced VO_2_ responsiveness (yes or no). The DeLong's test was used to determine the difference in AUC between subgroups with a minimum calculated sample size (16).

The Power Analysis and Sample Size software (NCSS, LLC, Kaysville, UT, USA) was utilized to determine the statistical power. Previous studies indicated that both *Δ*ScvO_2_ and *Δ*P(cv–a)CO_2_ had an area under the receiver operating characteristic (ROC) curve (AUC) of approximately 0.8 (2, 3). To achieve a power of 80% with an alpha risk of 0.05, it was determined that 26 subjects would be sufficient. Therefore, at least 52 patients were required to ensure adequate statistical power for each arm in the subgroup analyses. In addition, we randomly selected 10 patients to calculate the coefficient of variation (CV) and the least significant change (LSC) to assess the intra-operator reproducibility for VTI. A two-tailed *P*-value of less than 0.05 was considered statistically significant.

## Results

A total of 58 consecutive patients were enrolled over one year, and 30 (51.7%) of them were classified as fluid responders (Figure 1). Distributive shock represented the primary cause of hypotension in this study (79.3%, 46/58), and the baseline characteristics and clinical outcomes were comparable between the responders and non-responders. Of note, all patients but one received norepinephrine infusion during the study period, and the intra-operator reproducibility for VTI was deemed acceptable with a CV of 4.0% [95% confidence interval (CI): 1.4%–6.6%] and a LSC of 6.4% (95% CI: 2.2%–10.5%). Table 1 presents the baseline characteristics of the patients.

**Table 1:** Baseline characteristics.

Variables	Responders (*n* = 30)	Non-responders (*n* = 28)	*P-*value
Age (years), median (IQR)	73 (56, 78)	71 (56, 78)	0.864
Male, *n* (%)	18 (60.0)	18 (64.3)	0.791
Body mass index (kg/m^2^), mean ± SD	22.5 ± 3.8	24.0 ± 3.6	0.136
Concurrent diseases, *n* (%)			
Hypertension	14 (46.7)	20 (71.4)	0.056
Diabetes	8 (26.7)	6 (21.4)	0.641
Coronary heart disease	7 (23.3)	4 (14.3)	0.380
Chronic kidney disease	2 (6.7)	3 (10.7)	0.665
Causes of hypotension, *n* (%)			
Distributive	23 (76.7)	23 (82.1)	0.607
Hypovolemic	5 (16.7)	2 (7.1)	0.425
Cardiogenic	2 (6.7)	3 (10.7)	0.665
Tidal volume (mL/kg of PBW), mean ± SD	8.0 ± 1.9	7.8 ± 1.3	0.684
Driving pressure (cmH_2_O), median (IQR)	12 (10, 13)	13 (10, 14)	0.334
PEEP (cmH_2_O), median (IQR)	5 (5, 8)	5 (5, 8)	0.745
FiO_2_ (%), median (IQR)	45 (39, 50)	40 (31, 60)	0.370
Respiratory rate (breaths/min), median (IQR)	17 (15, 22)	16 (15, 18)	0.663
Analgesia and sedation, *n* (%)			
Midazolam	19 (63.3)	21 (75.0)	0.337
Propofol	11 (36.7)	8 (28.6)	0.512
Fentanyl	8 (26.7)	10 (35.7)	0.457
Butorphanol	9 (30.0)	8 (28.6)	0.905
APACHE II score, mean ± SD	19 ± 6	21 ± 5	0.197
SOFA score, mean ± SD	9 ± 3	10 ± 3	0.094
Dose of norepinephrine (*μ*g/kg/min), median (IQR)	0.24 (0.10, 0.34)	0.22 (0.17, 0.27) (n = 27)	0.879
Inotropic agents, *n* (%)	9 (30.0)	10 (35.7)	0.643
Duration of invasive mechanical ventilation (days), median (IQR)	11 (5, 18)	9 (4, 17)	0.858
Length of ICU stay (days), median (IQR)	13 (6, 24)	11 (6, 22)	0.767
ICU mortality, *n* (%)	8 (26.7)	5 (17.9)	0.421

SD, standard deviation; IQR, interquartile range; PBW, predicted body weight; APACHE, acute physiology and chronic health evaluation; SOFA, sequential organ failure assessment; PEEP, positive end-expiratory pressure; FiO_2_, fraction of inspired oxygen; ICU, intensive care unit.

### Hemodynamic changes induced by fluid expansion

Before the fluid challenge, no significant differences in the baseline hemodynamic variables were observed between the responders and non-responders. After the fluid challenge, SV, cardiac index, and DO_2_ were remarkably increased in the responders, but not in the non-responders. Hemoglobin was significantly decreased after fluid expansion in both groups. In the responders, volume expansion led to an elevated ScvO_2_ and a reduced P(cv–a)CO_2_, whereas these values remained unchanged in the non-responders. Table 2 shows the fluid-induced changes in hemodynamic variables in detail.

**Table 2:** Changes in hemodynamic variables induced by volume expansion.

Variables	Responders (*n* = 30)		Non-responders (*n* = 28)		*P-*value^a^	*P-*value^b^
	Before	After	Before	After		
HR (beats/min)	88 (74, 112)	91 (78, 110)	95 (85, 125)	95 (89, 113)	0.085	0.181
CVP (mmHg)	9 (7, 11)	11 (10, 13)*	11 (8, 14)	15 (12, 17)*	0.093	0.002
SAP (mmHg)	108 ± 14	116 ± 17*	102 ± 11	115 ± 15*	0.098	0.736
DAP (mmHg)	55 ± 8	62 ± 10*	57 ± 7	58 ± 7	0.243	0.096
MAP (mmHg)	71 ± 8	80 ± 11*	72 ± 6	76 ± 7*	0.588	0.067
SV (mL)	48 ± 10	60 ± 13*	50 ± 13	50 ± 14	0.523	0.012
Cardiac index (L/min/m^2^)	2.60 (2.25, 3.03)	3.35 (2.78, 4.03)*	2.70 (2.23, 3.30)	2.80 (2.23, 3.40)	0.523	0.017
Hemoglobin (g/L)	113 ± 25	104 ± 24*	100 ± 30	96 ± 27*	0.082	0.236
Arterial PH	7.39 ± 0.10	7.38 ± 0.09	7.40 ± 0.06	7.39 ± 0.06*	0.423	0.468
PaCO_2_	40 (34, 43)	40 (37, 43)	38 (33, 45)	38 (34, 43)	0.749	0.468
SaO_2_ (%)	99 (97, 100)	99 (98, 100)	99 (98, 100)	99 (98, 100)	0.345	0.743
Arterial lactate	1.5 (1.2, 2.0)	1.3 (0.9, 1.9)*	1.6 (1.0, 2.6)	1.6 (1.0, 2.4)*	0.668	0.450
Central venous PH	7.34 ± 0.10	7.33 ± 0.09*	7.36 ± 0.07	7.35 ± 0.06	0.463	0.311
PcvCO_2_	48 ± 10	46 ± 8*	46 ± 9	46 ± 8	0.371	0.784
ScvO_2_ (%)	66 ± 15	72 ± 13*	68 ± 14	68 ± 14	0.571	0.365
P(cv-a)CO_2_	8 (6, 10)	6 (5, 8)*	6 (4, 9)	7 (5, 10)	0.135	0.551
VO_2_ (mL/min)	159 (120, 270)	172 (122, 259)	168 (112, 250)	139 (111, 236)	0.756	0.335
DO_2_ (mL/min)	609 (497, 759)	723 (600, 880)*	683 (450, 754)	612 (444, 758)	0.803	0.029

All data were presented as mean ± standard deviation or median with interquartile range.

HR, heart rate; CVP, central venous pressure; SAP, systolic arterial pressure; DAP, diastolic arterial pressure; MAP, mean arterial pressure; SV, stroke volume; PH, potential of hydrogen; PaCO_2_, arterial partial pressure of carbon dioxide; SaO_2_, arterial oxygen saturation; PcvCO_2_, central venous partial pressure of carbon dioxide; ScvO_2_, central venous oxygen saturation; P(cv-a) CO_2_, central venous to arterial carbon dioxide tension difference; VO_2_, consumption of oxygen; DO_2_, oxygen delivery.

a *P*-value for the comparison between responders and non-responders before volume expansion;

b *P*-value for the comparison between responders and non-responders after volume expansion;

* *P* < 0.05 for the intra-group comparison before vs. after volume expansion.

### Relationship between fluid responsiveness and *Δ*ScvO_2_, and *Δ*p(cv–a)CO_2_

**Figure 2:**
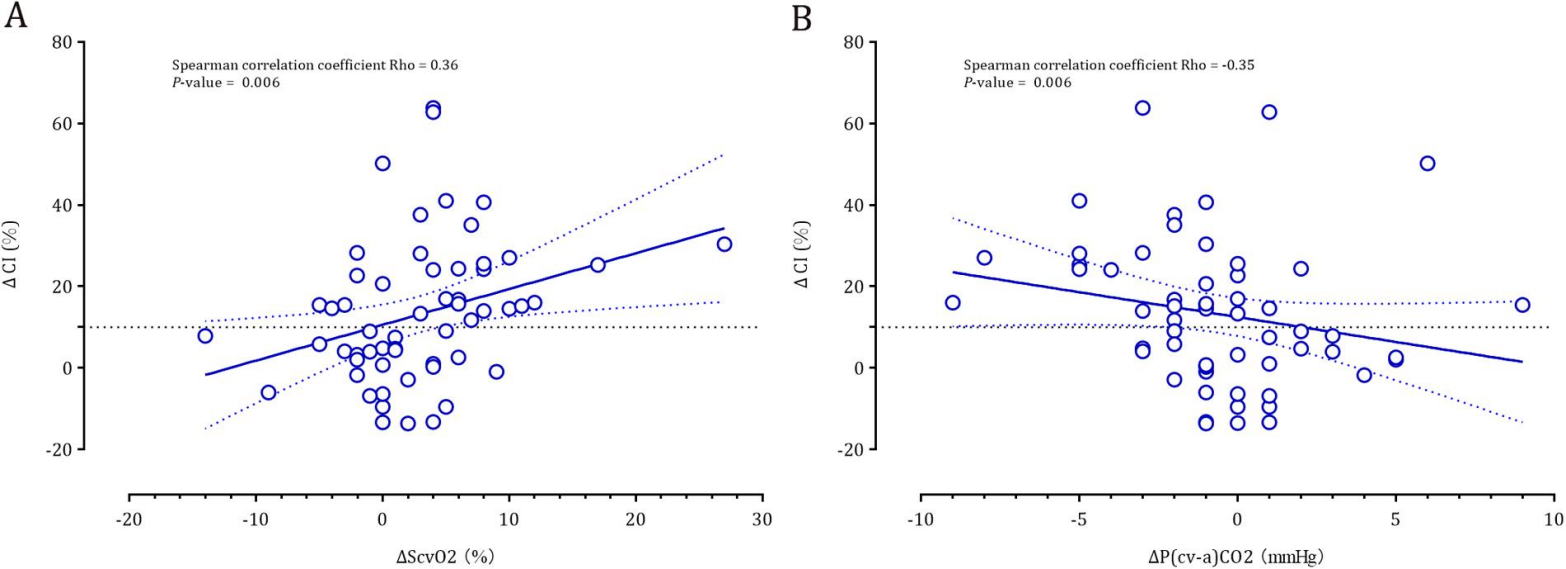
Correlation between the fluid-induced cardiac index and *Δ*ScvO_2_ (panel **A**) and *Δ*P(cv-a)CO_2_ (panel **B**).

Spearman correlation analyses revealed that the fluid-induced changes in cardiac index were positively correlated with *Δ*ScvO_2_ (rho = 0.36, *P* = 0.006) and were negatively correlated with *Δ*P(cv–a)CO_2_ (rho = −0.35, *P* = 0.006) (Figure 2).

**Figure 3:**
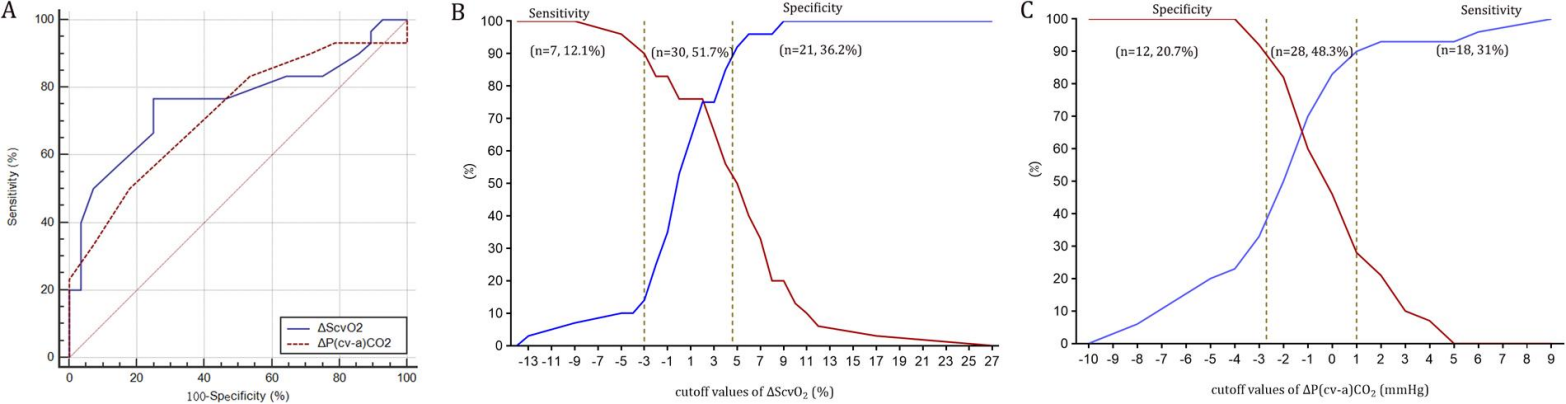
Receiver operating characteristic curves (panel **A**) and the gray zone for *Δ*ScvO_2_ (panel **B**) and *Δ*P(cv-a)CO_2_ (panel **C**).

*Δ*ScvO_2_ and *Δ*P(cv-a)CO_2_ defined fluid responsiveness with AUC values of 0.76 (95% CI: 0.63–0.86; *P* < 0.001) and 0.72 (95% CI: 0.59–0.83; *P* < 0.001) (Figure 3), respectively. Based on the Youden index, the optimal cutoff value of *Δ*ScvO_2_ was 2%, with a sensitivity of 76.7% and a specificity of 75.0%. However, considering the repeatability of ScvO_2_ (a SDD of ±3%), the optimal cutoff value was 5%, yielding a sensitivity of 50%, a specificity of 92.9%, a positive predictive value (PPV) of 88%, and a negative predictive value (NPV) of 63%. The gray zone approach identified a *Δ*ScvO_2_ range of −3% to 4.6%, which included 51.7% of the patients (Figure 3). According to the Youden index, the optimal cutoff value of *Δ*P(cv-a)CO_2_ was −2 mmHg, which exceeded the SDD of P(cv-a)CO_2_ (±2 mmHg). Thus, the optimal cutoff value of −2 mmHg yielded a sensitivity of 50%, a specificity of 82.1%, a PPV of 75%, and an NPV of 61%. A range of −2.7 mmHg to 1 mmHg represented the gray zone for *Δ*P(cv-a)CO_2_ that comprised 48.3% of patients (Figure 3).

### Subgroup analyses

Given the minimum calculated sample size, the findings from the subgroup analysis with a sample size of 26 cases or more were considered statistically valid. Subgroup analyses demonstrated that *Δ*ScvO_2_ potentially exhibited better accuracy for assessing fluid responsiveness in VO_2_ non-responders and patients with a baseline ScvO_2_ < 70%. Meanwhile, the diagnostic accuracy of *Δ*P(cv-a)CO_2_ was slightly improved in VO_2_ non-responders and patients with a baseline P(cv-a)CO_2_ ≥ 6 mmHg (Table 3). The AUC of *Δ*ScvO_2_ in the subgroup with a baseline ScvO_2_ < 70% was slightly higher than that in the subgroup with a baseline ScvO_2_ ≥ 70%, despite no statistical significance (*P* = 0.219). However, the comparisons of AUC in other subgroups were not conducted because the minimum sample size in some subgroups was not reached.

**Table 3:** Subgroup analyses for diagnostic accuracies of *Δ*ScvO_2_ and *Δ*P(cv-a)CO_2_ in assessing fluid responsiveness.

Variables	AUC	*P*-value	Cutoff value^a^	Sensitivity (%)	Specificity (%)
*Δ*ScvO_2_ (%) (*n* = 58)	0.76 (0.63, 0.86)	<0.001	2	76.7 (57.7, 90.1)	75.0 (55.1, 89.3)
VO_2_ non-responders (*n* = 40)	0.91 (0.78, 0.98)	<0.001	2	100 (82.4, 100)	66.7 (43.0, 85.4)
VO_2_ responders (*n* = 18)	–	–	–	–	–
Baseline ScvO_2_ ≥ 70% (*n* = 26)	0.67 (0.46, 0.84)	0.147	2	53.8 (25.1, 80.8)	100 (75.3, 100)
Baseline ScvO_2_ < 70% (*n* = 32)	0.84 (0.67, 0.95)	<0.001	5	70.6 (44.0, 89.7)	86.7 (59.5, 98.3)
*Δ*P(cv-a)CO_2_ (mmHg) (*n* = 58)	0.72 (0.59, 0.83)	<0.001	−2	50.0 (31.3, 68.7)	82.1 (63.1, 93.9)
VO_2_ non-responders (*n* = 40)	0.78 (0.62, 0.90)	<0.001	−2	63.2 (38.4, 83.7)	81.0 (58.1, 94.6)
VO_2_ responders (*n* = 18)	–	–	–	–	–
Baseline P(cv-a)CO_2_ ≥ 6 mmHg (*n* = 39)	0.78 (0.62, 0.90)	<0.001	−2	65.2 (42.7, 83.6)	75.0 (47.6, 92.7)
Baseline P(cv-a)CO_2_ < 6 mmHg (*n* = 19)	–	–	–	–	–

Data were not presented when the sample size were less than 26 cases.

*Δ*ScvO_2_, the variation in central venous oxygen saturation; *Δ*P(cv-a)CO_2_, the variation in central venous to arterial carbon dioxide tension difference; VO_2_, consumption of oxygen; AUC, area under the receiver operating characteristic curve.

a Cutoff value determined by identifying the maximum from the Youden index.

## Discussion

In this perspective observational study, the principal findings demonstrated that *Δ*ScvO_2_ and *Δ*P(cv-a)CO_2_ during volume expansion possessed an acceptable diagnostic accuracy for identifying fluid responsiveness, and the diagnostic accuracies of *Δ*ScvO_2_ and *Δ*P(cv-a)CO_2_ were likely associated with their baseline values and the fluid-induced VO_2_ responsiveness.

Consistent with the optimal CI of *Δ*ScvO_2_ (3% to 5%) identified in our recent meta-analysis (3), we determined the optimal cutoff value for *Δ*ScvO_2_ as 5% in the current study, yielding a high PPV (88%) and a relatively low NPV (63%). Thus, we can almost confirm that a patient can benefit from volume expansion if the measured *Δ*ScvO_2_ is greater than 5%. However, we cannot make any decisions if the measured *Δ*ScvO_2_ is less than 5%, due to the low NPV. Indeed, a low *Δ*ScvO_2_ does not necessarily indicate a small change in CO induced by fluid challenge. According to the Fick principle, the close relationship between ScvO_2_ and CO depends on stable oxygen content and VO_2_ during volume expansion (7). However, a potential decrease in Hb concentration could somewhat reduce oxygen content. We observed a median reduction in Hb of 5.9% after volume expansion across the entire population studied, which was consistent with a recent meta-analysis (17). Furthermore, VO_2_ does not always remain constant during volume expansion because of the VO_2_/DO_2_ dependency phenomenon. The VO_2_/DO_2_ dependency phenomenon refers to a linear correlation between DO_2_ and VO_2_ when DO_2_ decreases below the critical value (18), which implies that VO_2_ will change linearly with DO_2_, thus resulting in a relatively constant oxygen extraction and ScvO_2_ (3). In these situations, ScvO_2_ would not change significantly (i.e., a low *Δ*ScvO_2_) despite a noticeable increase in CO and DO_2_. This could explain why the diagnostic accuracy of *Δ*ScvO_2_ was improved considerably after excluding the VO_2_ responders. Additionally, subgroup analysis revealed that the AUC of *Δ*ScvO_2_ was increased after excluding patients with a baseline ScvO_2_ ≥ 70% (see Table 3), which suggested that the baseline ScvO_2_ may be a determinant of the diagnostic accuracy of *Δ*ScvO_2_, even though the baseline ScvO_2_ seems unable to identify fluid responsiveness (2, 19). Indeed, a normal or supranormal ScvO_2_ value typically indicates an adequate CO to provide sufficient oxygen delivery and/or mitochondrial dysfunction or microcirculatory shunting. In this case, the magnitude of *Δ*ScvO_2_ induced by volume expansion may be limited and may not be parallel to the fluid-induced increases in CO.

In addition, we also confirmed the ability of *Δ*P(cv-a)CO_2_ to define fluid responsiveness. However, the diagnostic accuracy of *Δ*P(cv-a)CO_2_ (AUC of 0.72) in our study appears to be lower than that in a previous study (AUC of 0.831) (2). This discrepancy is not surprising given the complex relationship between *Δ*P(cv-a)CO_2_ and CO. It should be recognized that the close association between *Δ*P(cv-a)CO_2_ and CO relies on a stable CO_2_ content-CO_2_ partial pressure relationship, as well as a stable relationship between P(cv-a)CO_2_ and CO (5, 20). However, the curvilinear CO_2_ content-CO_2_ partial pressure relationship can be influenced by metabolic acidosis, hematocrit, or the Haldane effect, which refers to the effect of oxygen saturation on CO_2_ transport (5, 20). Consequently, varying baseline values for these variables may result in differing diagnostic accuracies of *Δ*P(cv-a)CO_2_ across various studies. Furthermore, the relationship between P(cv-a)CO_2_ and CO is also curvilinear. This means that for a constant total CO_2_ production, fluid-induced changes in CO can cause a more significant alteration in P(cv-a) CO_2_ at a low CO value than at a normal or high CO value (5). This may explain why *Δ*P(cv-a)CO_2_ was more effective in defining fluid responsiveness in patients with a baseline P(cv-a)CO_2_ ≥ 6 mmHg, as a high P(cv-a)CO_2_ level typically indicates a low baseline CO. Similarly, a previous study found that fluid-induced CO increases engendered a reduction in P(cv-a) CO_2_ only in patients with elevated baseline P(cv-a)CO_2_ values (≥6 mmHg), but not in those with normal baseline levels (21). The subgroup analysis also revealed an improved diagnostic accuracy of *Δ*P(cv-a)CO_2_ when excluding patients with VO_2_ responsiveness. This finding aligns with the study conducted by Nassar et al. (4). Specifically, CO_2_ production associated with anaerobic metabolism tends to occur at the VO_2_/DO_2_ dependency stage (5, 6). This phenomenon may attenuate the relationship between *Δ*P(cv-a)CO_2_ and fluid-induced CO increases.

Our findings provide a clinical implication: when the CO measurement is not available, measuring ScvO_2_ or P(cv-a)CO_2_ before and after volume expansion can help identify which patients are likely to benefit from fluid therapy, particularly for patients with abnormal baseline values or with VO_2_ unresponsiveness. However, several limitations in this study should be highlighted. First, the limited sample size in this study could overestimate the effect sizes, especially hampering us from drawing a firm conclusion in the subgroup analysis. Second, ScvO_2_ was measured in this study instead of the mixed venous oxygen saturation. As ScvO_2_ primarily reflects the DO_2_-VO_2_ relationship in the upper side of the body, it may not inform about the local perfusion disturbances in regional septic conditions (5). Despite this, the changes in ScvO_2_ can track the global DO_2_ changes, given the equivalent changing trend of ScvO_2_ and the mixed venous oxygen saturation (22). Finally, there may be mathematical coupling issues in the estimation of DO_2_ and VO_2_ based on the Fick method, which may introduce bias in the results of subgroup analysis.

## Conclusion

In mechanically ventilated patients, *Δ*ScvO_2_ and *Δ*P(cv-a)CO_2_ induced by volume expansion are potential indicators for assessing fluid responsiveness and may be routinely measured to indicate fluid responsiveness in the absence of CO measurement. The diagnostic accuracies of *Δ*ScvO_2_ and *Δ*P(cv-a)CO_2_ were likely associated with the baseline ScvO_2_ and P(cv-a)CO_2_ values and the fluid-induced VO_2_ responsiveness.

## Data Availability

The original contributions presented in the study are included in the article/Supplementary Material, further inquiries can be directed to the corresponding authors.
